# Individuals with Mental Illness and Stigma Reduction: A Cross-Sectional Study in a Group of College Students

**Published:** 2019-10

**Authors:** Abdulaziz Aflakseir, Muhammad Rasooli Esini, Muhammad Goodarzi, Javad Molazadeh

**Affiliations:** Department of Clinical Psychology, School of Education and Psychology, Shiraz University, Shiraz, Iran,

**Keywords:** *Contact*, *College Students*, *Dangerousness*, *Mental Illness*, *Stigmatizing Attitude*

## Abstract

**Objective:** Stigma has a significant impact on the life of individuals with mental illness. The purpose of this study was to examine the association of contact with the mentally ill with stigmatizing attitudes in a group of college students.

**Method** **:** A total of 287 college students participated in this study. The participants were recruited from Hormozgan University of Medical Sciences using convenience sampling and completed the research measures including the Level of Familiarity (LOF) and the Attribution questionnaires (AQ). The data were analyzed using SPSS.

**Results: **The descriptive findings of this study showed that the participants’ highest score on stigmatizing attitudes was related to pity and the least score was related to anger towards people with mental illness. Furthermore, the regression analysis results indicated that personal contact, family contact, and work contact with individuals with mental illness significantly predicted stigma reduction, while other types of contacts with the mentally ill, such as friend contact, social contact, and media contact, did not significantly predict stigma reduction.

**Conclusion: **This study highlighted the significant role of having contact with the mentally ill in reducing stigmatizing attitudes towards them.

One of the most marked out, denounced, and labeled group of the society are the individuals with mental illnesses, as they experience humiliation, unfairness, and prejudice (1). Stigma occurs in form of disgrace and dishonor imposed from the society and can have a negative impact on treatment, occupation, profession, and self-esteem (2). The word stigma is described as labeling, discrimination, and rejection of individuals who are behaviorally and socially different (3). Individuals who suffer from mental illness are usually labeled due to some reasons, such as their behavior and the negative image of psychiatric disorders depicted in the media (4). Persons with mental disorders are viewed to be dangerous, violent, and threatening. The stigma is not only experienced by the individual but it can also impact the family of that person. Research suggests that direct contact with individuals with mental illness can decrease the aforementioned negative views towards them (5). Results of previous studies revealed that acquainting with someone suffering from mental illness leads to a more positive viewpoint of these individuals and increases the willingness to have contact with them (6, 7). Both personal and professional contact with people suffering from mental illnesses have been reported to diminish stigma. Some studies showed that people who have acquaintance with mentally ill individuals consider these people as less dangerous in general and are ready to have social contact with them (7). Based on research on the sociodemographic aspects, older age and lower education level are 2 factors that influence negative attitudes towards mental disorders (8).

In a survey on stigmatization in 27 countries, it was found that 50% of people with mental disorders reported discrimination in their personal relationships, and three-quarters anticipated discrimination while applying for work (9). Similarly, in a study in India, a high level of stigma was reported among people (10). In a study with a large sample size in Tehran, it was found that about 52% of the general public believed that individuals with mental disorders are dangerous. 

Furthermore, 17% of participants were afraid of having conversation with patients suffering from mental illness (11). Although several studies have been conducted on stigmatizing attitudes of Iranian general public towards mentally ill individuals, there is little information on the association between contact with people suffering from mental illnesses and stigmatizing attitudes in Iranian society. Furthermore, many studies have indicated that stigmatization attitude is influenced by cultural factors. Therefore, it is important to understand stigmatization within the cultural context of Iranian society. The aim of this study was to examine the association between the various types of contact (personal, family, friend, work, social, and media) with the mentally ill and stigmatizing attitudes among a group of college students.

## Materials and Methods

***Research Design***

This was a descriptive and cross-sectional study. The various levels of contact with mentally ill individuals were considered as the independent variables and the stigmatizing attitude of dangerousness was regarded as the dependent variable.

***Participants***

A total of 287 college students took part in this study (175 women and 112 men). Participants were recruited from Hormozgan University of Medical Sciences using convenience sampling method. The sample included students from several faculties of Hormozgan University of Medical Sciences, including Nursing and Midwifery, Allied Medical Sciences, and Health. The inclusion criterion was willingness to participate in the study. The inclusion criterion was having a history of mental illness.

***Procedure***

The participants were also asked to complete the demographics questionnaire and specify their age, level of education, marital status, and gender. The second author of this article distributed the questionnaires among the college students in class. 

***Instruments***

Level of Familiarity (LOF): The LOF was developed by Holemes et al (12). This scale consists of 12 items on varying degrees of intimate contact with a person with mental illness. Each item is rated on an 11-point scale from 1 (lowest level of contact) to 11 (highest level of contact). The questionnaire included six topics, including personal, family, friend, work, media, and social contact. The items were divided into 6 two-item dimensions. The author reported a good reliability and validity for this scale, with Cronbach's alpha of 0.83 (12). The present study showed a good reliability for the scale. The internal consistency was calculated and the findings showed a Cronbach's alpha of 0.80. Furthermore, the LOF was validated in this study.

Attribution Questionnaire (AQ): The AQ has been developed to assess stigmatization (13). Before completing the scale, the participants are provided with a short and neutral statement about someone who works as a staff in a company and has been admitted to a hospital for schizophrenia (13). The AQ consists of 9 subscales and has 27 items. For the purpose of this study, only one component of this scale (dangerousness) was used to assess stigmatizing attitude. Then, the answers were rated on a 9-point Likert scale from 1 (none) to 9 (very much), with higher scores indicating greater perceived dangerousness of and distant from individuals with mental illness. Studies have reported good validity and reliability for this scale (14). This scale has been utilized in Iran and found to have acceptable reliability, with Cronbach alpha coefficient of 0.92. Tavakoli et al have also reported a good validity for the LOF (15). 

***Statistical Analysis***

The statistical analyses, such as mean, standard deviation, Pearson correlation coefficient, and simultaneous multiple regression analysis, were used to analyze the data using the SPSS software (version 20). Multiple regression analysis was used to examine the prediction of stigmatizing attitude (dangerousness) based on the various types of contact with mentally ill persons. The p value of 0.05 was considered as significant in this study.

***Ethics***

The research proposal was approved by the research ethics committee of Hormozgan University of Medical Sciences. The participants were briefed about the study and were informed of their right to leave the study at any time. All information was kept confidential and consent form was obtained from each participant.

## Results

The mean age of participants was 22.10 years, ranging from 18 to 25 years (SD = 4.12). Most participants were female (60.9%) and single (84.7%) and about 79% were undergraduate. The demographic data are presented in Table 1 and descriptive data in Table 2. The highest score on stigmatizing attitudes was for pity (M = 20.54, SD = 5.08), followed by help (M = 19.75, SD = 5.02), and the least score was related to anger (M = 8.98, SD = 3.21) (Table 2). In other words, the participants felt pity towards individuals with mental illness and had less anger towards them. In terms of the relationship between the various types of contact and mentally ill individuals and stigmatization, the results revealed a significant association between family contact (r = -0.25, p < 0.01), personal contact (r = -0.23, p < 0.01), work contact (r = -0.19, p < 0.01), and stigma reduction. In other words, those who had a family member with mental illness, had a direct experience with the mentally ill, and were familiar with individuals with mental disorders at work had less stigmatizing attitudes. The strongest association was between family contact with the mentally ill and stigma reduction. The data are presented in Table 3. Findings of the regression analysis revealed that personal contact (ß = -0.28, p < 0.03), family contact (ß = -0.32, p < 0.01), and work contact (ß = -0.24, p < 0.05) significantly predicted less stigmatizing attitude. The family contact with the mentally ill was the strongest variable that predicted stigma reduction. The model explained 23% of the variance in stigmatization (R2 = 0.23). The results of multiple regression analysis are presented in Table 4. The findings of this study revealed that the greater the amount of contact the college students had with the mentally ill, the lower stigma they showed.

**Table 1:** Demographics Characteristics of Participants (n = 287)

**Variable**	**Frequency**	**percent**
Age Mean (SD)	22.6(4.12)	
**Gender**		
Male	112	39.03
Female	175	60.97
**Education**		
Undergraduate	226	78.74
Postgraduate	37	13.89
Medical	24	8.36
**Marital Status**		
Married	44	15.28
Single	243	84.72

**Table 2:** Descriptive statistics for Participants on the Dimensions of the Attribution Questionnaire

**Stigmatization ** **Dimension**	**M**	**SD**
Blame	12.32	3.97
Pity	20.54	5.08
Anger	8.98	3.21
Dangerousness	12.82	4.01
Fear	11.83	3.60
Help	20.06	5.02
Avoidance	17.76	5.20

M=Mean; SD=Standard Deviation

**Table 3:** Relationship between the Various Types of Contact with the Mentally Ill and Stigmatization (Dangerousness)

**The Types of Contact**	**r**	**p**
Personal Contact	-0.23	0.01
Work Contact	-0.20	0.01
Family Contact	-0.25	0.01
Social Contact	-0.10	0.09
Media Contact	0.07	0.12
Friend Contact	-0.08	0.10

**p<0.01, *p<0.05

**Table 4:** Multiple Regression Analysis for Predicting Stigmatizing Attitudes (Dangerousness) (n = 287)

**Variable**	**B**	**SE**	**β**	**t**	***p***
Work Contact	-2.13	1.23	-0.24	-1.45	0.05
Friends Contact	-0.95	0.98	-0.10	-1.10	0.09
Family Contact	-1.35	0.25	-0.32	-1.95	0.01
Social Contact	0.87	0.45	-0.09	-0.78	0.11
Personal Contact	-1.64	0.15	-0.28	-1.76	0.03
Media contact	-1.02	0.58	-0.07	-0.35	0.32

B= Unstandardized coefficient, SE= Standard Error, ß = Beta, t = t test

## Discussion

The present study aimed to examine the stigmatization and its association with the level of contact with individuals suffering from mental illness. Descriptive findings showed that the participants felt a high level of pity for the mentally ill. There are mixed findings on the effect of pity on the mentally ill. Some surveys have reported that pity on psychiatric patients has positive effects, such as providing resources for mental health plans (16). On the other hand, most studies have concluded that pity has a negative impact on these people. They argue that pity decreases the empowerment and self-esteem of these patients (17, 18). The high sympathy of the participants may reflect that Iranians think it is kind and helpful to show pity to individuals with disabilities and mental illness. The participants who felt pity for the mentally ill were also more willing to offer them help (18). The relatively high score of the participants on help dimension of stigmatization confirmed the literature that indicated viewing people with mental disorders pitifully brings them help by the general public. The findings on stigmatization dimensions such as anger, fear and dangerousness were also similar to other research showing that educated individuals have a positive view about persons with mental illness (18). The results of this study on the relationship between interpersonal contact and stigmatizing attitudes revealed that certain types of contact, such as family, personal, and work contact with the mentally ill reduced stigma towards these individuals. Also, the results provided more support for the importance of contact in reducing stigma. The findings of this study are in agreement with those of Reinke’s research (2004), indicating that a lower level of contact was associated with higher level of stigmatization (19). Similar results were reported in a study conducted by Corrigan and Penn (13) that stigma decreased with contact. Based on a previous study, people who have experience of contact with mentally ill individuals perceive them more positively and are more likely to employ them (20). Also, the findings of this study are consistent with those of Corrigan’s study, showing that close contact with the mentally ill leads to less stigmatizing attitudes towards these persons (21). Similarly, the results of this study are in agreement with those of a study that concluded direct contact with individuals suffering from mental illness is one of the most effective ways to reduce stigma (22). Moreover, having acquaintance with individuals suffering from mental disorders has been proven to bring about more positive attitudes (23, 24). The present study also supports Alexander and Link’s study (2003) revealing that the more contact participants have with the mentally ill, the less dangerous they consider them to be (25). This study highlighted that closer contact of the college students with individuals with mental disorders were more likely to have less negative attitudes and stigma towards the mentally ill. According to findings of the current study, other types of contact, such as social, friends, and media, did not have a significant role on stigmatization, which may be due to the fact that participants who knew patients in this context, had less chance to have an intimate contact with individuals suffering from mental illness. The health system should provide education to the public to diminish misconception about mental illness and improve attitudes towards individuals with mental illness.

## Limitation

This study had several limitations that must be noted. First, the study used self-report measures with no observational or interview data. The second limitation was related to sample. The sample of this study was not diverse in terms of demographic and socioeconomic factors. Thus, future studies should select participants with various demographics and socioeconomic status. In addition, the present study did not examine the impact of some other significant variables, such as the type of mental illness, demographic factors, and socioeconomic status, on stigmatization. This research highlighted the importance of close contact with individuals suffering from mental disorders to reduce stigma towards them.

## Conclusion

The present study showed that those college students who had a closer contact with people with mental disorders had less stigmatizing attitudes towards these individuals. This study also highlighted the importance of contact with mentally ill people for reducing people’s negative attitudes towards persons with mental illness.
